# Cancer and Infective Endocarditis: Characteristics and Prognostic Impact

**DOI:** 10.3389/fcvm.2021.766996

**Published:** 2021-11-11

**Authors:** Bernard Cosyns, Bram Roosens, Patrizio Lancellotti, Cécile Laroche, Raluca Dulgheru, Valentina Scheggi, Isidre Vilacosta, Agnès Pasquet, Cornelia Piper, Graciela Reyes, Essam Mahfouz, Zhanna Kobalava, Lionel Piroth, Jarosław D. Kasprzak, Antonella Moreo, Jean-François Faucher, Julien Ternacle, Marwa Meshaal, Aldo P. Maggioni, Bernard Iung, Gilbert Habib

**Affiliations:** ^1^Centrum Voor Hart- en Vaatziekten (CHVZ), Vrije Universiteit Brussel (VUB), Universitair Ziekenhuis Brussel (UZ Brussel), Brussels, Belgium; ^2^In vivo Cellular and Molecular Imaging (ICMI) Center, Vrije Universiteit Brussel (VUB), Brussels, Belgium; ^3^Cardiology Department, University Hospital Centre, Centre Hospitalier Universitaire (CHU) Sart Tilman, Liège, Belgium; ^4^EURObservational Research Programme, European Society of Cardiology, Antibes, France; ^5^Cardiothoracic and Vascular Department, Careggi University Hospital, Florence, Italy; ^6^Department of Cardiology, Hospital Clínico San Carlos, Universidad Complutense de Madrid, Madrid, Spain; ^7^Pôle de Recherche Cardiovasculaire, Institut de Recherche Expérimentale et Clinique, Université Catholique de Louvain, Brussels, Belgium; ^8^Divisions of Cardiology and Cardiothoracic Surgery, Cliniques Universitaires Saint-Luc, Brussels, Belgium; ^9^Clinic for General and Interventional Cardiology/Angiology, Herz- und Diabeteszentrum Nordrhein-Westfalen (NRW), Ruhr-Universität Bochum, Bad Oeynhausen, Germany; ^10^Echo Lab Department, Hospital de Alta Complejidad en red El Cruce, Florencio Varela, Buenos Aires, Argentina; ^11^Mansoura Specialized Medical Hospital, Mansoura University, Mansoura, Egypt; ^12^Department of Cardiology, Rossiyskiy Universitet Druzhby Narodov (RUDN) University Moscow, Moscow, Russia; ^13^Infectious Diseases Department, University Hospital, INSERM CIC1432, University of Burgundy, Dijon, France; ^14^Bieganski Hospital, Medical University of Lodz, Łódź, Poland; ^15^A. De Gasperis Cardio Center, ASST Grande Ospedale Metropolitano Niguarda, Milan, Italy; ^16^CHU Limoges, Department of Infectious Diseases and Tropical Medicine, Limoges France INSERM, U1094, Limoges, France; ^17^Cardiology Department, Henri Mondor Hospital, SOS Endocardites, DHU ATVB, INSERM U955 Team 8, Université Paris-Est Créteil, Créteil, France; ^18^Fellow Equipe, Team du Dr Philippe Pibarot, Institut Universitaire de Cardiologie et de Pneumologie de Québec/Quebec Heart and Lung Institute Université Laval, Quebec City, QC, Canada; ^19^Cardiology Department, Kasr Al Ainy, Cairo University, Cairo, Egypt; ^20^Maria Cecilia Hospital, Gruppo Villa Maria (GVM) Care & Research, Cotignola, Italy; ^21^Bichat Hospital, APHP, DHU Fire, Paris Diderot University, Paris, France; ^22^AP-HM, La Timone Hospital, Cardiology Department, Marseille, France; ^23^Aix Marseille University, IRD, AP-HM, MEPHI, IHU-Mediterranean Infection, Marseille, France

**Keywords:** cancer, cardiac surgery, infective endocarditis, registry, valve disease

## Abstract

**Background:** The interplay between cancer and IE has become of increasing interest. This study sought to assess the prevalence, baseline characteristics, management, and outcomes of IE cancer patients in the ESC EORP EURO-ENDO registry.

**Methods:** Three thousand and eighty-five patients with IE were identified based on the ESC 2015 criteria. Three hundred and fifty-nine (11.6%) IE cancer patients were compared to 2,726 (88.4%) cancer-free IE patients.

**Results:** In cancer patients, IE was mostly community-acquired (74.8%). The most frequently identified microorganisms were *S. aureus* (25.4%) and Enterococci (23.8%). The most frequent complications were acute renal failure (25.9%), embolic events (21.7%) and congestive heart failure (18.1%). Theoretical indication for cardiac surgery was not significantly different between groups (65.5 vs. 69.8%, *P* = 0.091), but was effectively less performed when indicated in IE patients with cancer (65.5 vs. 75.0%, *P* = 0.002). Compared to cancer-free IE patients, in-hospital and 1-year mortality occurred in 23.4 vs. 16.1%, *P* = 0.006, and 18.0 vs. 10.2%; *P* < 0.001, respectively. In IE cancer patients, predictors of mortality by multivariate analysis were creatinine > 2 mg/dL, congestive heart failure and unperformed cardiac surgery (when indicated).

**Conclusions:** Cancer in IE patients is common and associated with a worse outcome. This large, observational cohort provides new insights concerning the contemporary profile, management, and clinical outcomes of IE cancer patients across a wide range of countries.

## Introduction

Infective endocarditis (IE) is a severe disease, associated with important morbidity and mortality (1–4). Some IE patients have active, previously diagnosed cancer. In other patients, IE might be a marker of a new, unsuspected neoplasia (5, 6). The interplay between cancer and IE has become of increasing interest (5, 7). Cancer patients may be at higher risk for IE, because of reduced immunity (e.g., due to antineoplastic therapy), central venous lines or portal catheters (8). Moreover, the clinical presentation of IE patients with cancer could be less specific. Additionally, therapeutic options might be limited, due to frailty and a potentially higher mortality risk in case of surgery.

The ESC EORP European Endocarditis (EURO-ENDO) registry is a multicentre, prospective, observational cohort study of IE patients at hospitals in Europe and ESC-affiliated/non-affiliated countries. The aim of EURO-ENDO is to investigate the care and outcomes of IE (9). This sub-analysis sought to assess the prevalence of cancer in IE patients and to determine baseline characteristics, management, and outcomes compared to IE patients that are free of cancer.

## Materials and Methods

### Study Design and Data Collection

The detailed methodology of the ESC EORP EURO-ENDO registry has been previously reported (9). Briefly, from 1 January 2016 to 31 March 2018, patients older than 18 years who presented with IE were included. Inclusion criteria were a diagnosis of definite IE (or possible IE, but considered and treated as IE) based on the ESC 2015 IE criteria (10). IE patients with previously diagnosed cancer were identified. Cancer was defined as a previous or active, solid tumor, or hematologic malignancy. Data were collected at inclusion and during hospitalization, including demographics, patient history, Charlson index, age, and comorbidities (11). Moreover, data were collected concerning clinical, biological, microbiological, and echocardiographic findings, use of other imaging techniques [computed tomography (CT) scan, 18F-FDG PET/CT, leucocyte scintigraphy], medical therapy, complications, theoretical indications for surgery and in-hospital mortality (9). This study complies with the Declaration of Helsinki. National coordinators, in conjunction with local centers managed the approvals of national or regional ethics committees or Institutional Review Boards, according to local regulations. Informed consent has been obtained from all subjects (or their legally authorized representative).

### Data Management and Statistical Analysis

Data were collected by the collecting officers at the participating sites and entered in an online electronic case report form (CRF). Data quality was monitored by the ESC EORP Registry Project and Data management teams. Data quality control followed a data validation plan defined by the Registry Executive Committee team in collaboration with the EORP team. The first author had full access to all the study data and takes responsibility for its integrity and the data analysis. Continuous variables are expressed as mean ± standard deviation or as median and interquartile range. Comparisons among groups have been performed using Kruskall Wallis test for non-parametric data. Categorical variables are expressed as frequency and percentages. Among-group 2 × 2 comparisons were made using Pearson's Chi-squared χ^2^-test or Fisher's exact test if any expected cell count was < 5. In other cases, the Monte-Carlo estimate of the exact *P*-value was used. Univariable analysis was applied to both continuous and categorical variables. Pairwise correlations between all candidate variables (variables with *P* < 0.10 in univariable) within the model were tested before proceeding to the multivariable model. In case of correlation, some criteria were not taken into account. Plots of the Kaplan–Meier curves have been used to assess survival and event-free survival. A backward multivariable Cox regression analysis has been performed to evaluate possible predictors of outcomes in cancer patients. A significance level of 0.05 was required to allow a variable to stay within the model. Some measures of model of fit have been considered: concordance and the Goodness of fit test proposed by May and Hosmer. In addition, the proportional hazard ratios assumptions were graphically verified with the Schoenfeld residuals test. All analyses were performed using SAS statistical software version 9.4 (SAS Institute, Inc., Cary, NC, USA).

## Results

Three thousand and eighty-five IE patients were included (12). Three hundred and fifty-nine (11.6%) IE patients with cancer were identified and compared to 2,726 (88.4%) IE patients without cancer. IE was definite in 304/359 (84.7%) and possible in 55/359 (15.3%) cancer patients. The age of and most frequent types of cancer can be found in Supplementary Table 1.

### Patient Demographics and Characteristics

The main demographic and characteristics of IE cancer patients are displayed in Table 1. IE was community-acquired in 74.8% and healthcare associated in 25.2% (nosocomial in 18.6%, non-nosocomial in 6.6%), native in 209 (60.4%), prosthetic in 97 (28.0%), device-related in 30 (8.7%), and repaired valve IE in 23 (2.9%) cancer patients. There were no significant differences with the cancer-free group. Valvular IE location was aortic in 52.7%, mitral in 47.0%, tricuspid in 5.7%, pulmonary in 0.9% of IE cancer patients. IE affected two or more valvular locations in 17.9%.

**Table 1:** Demographics and clinical characteristics of infective endocarditis patients.

	**Total**	**IE + cancer**	**IE – cancer**	***P*** **-value**
**Demography**				
*N*	3,085	359	2,726	
**Age (years)**				
Mean ± SD	59.21 ± 18.06	70.33 ± 11.47	57.74 ± 18.26	<0.001
Median (IQR)	63.0 (46.0–73.0)	72.0 (64.0–79.0)	61.0 (43.0–72.0)	<0.001
<65 years old	1,655/3,085 (53.6%)	90/359 (25.1%)	1,565/2,726 (57.4%)	<0.001
65–80 years old	1,060/3,085 (34.4%)	191/359 (53.2%)	869/2,726 (31.9%)	
≥80 years old	370/3,085 (12.0%)	78/359 (21.7%)	292/2,726 (10.7%)	
Females (%)	961/3,085 (31.2%)	110/359 (30.6%)	851/2,726 (31.2%)	0.824
**History of cardiovascular diseases**				
Heart failure	652/2,809 (23.2%)	75/307 (24.4%)	577/2,502 (23.1%)	0.592
Congenital heart disease	362/3,083 (11.7%)	11/359 (3.1%)	351/2,724 (12.9%)	<0.001
Ischemic heart disease	613/2,866 (21.4%)	89/318 (28.0%)	524/2,548 (20.6%)	0.002
Atrial fibrillation	756/2,887 (26.2%)	113/323 (35.0%)	643/2,564 (25.1%)	<0.001
Hypertrophic cardiomyopathy	63/2,809 (2.2%)	4/307 (1.3%)	59/2,502 (2.4%)	0.239
Known valve murmur	955/2,809 (34.0%)	97/307 (31.6%)	858/2,502 (34.3%)	0.347
Previous endocarditis (%)	271/3,085 (8.8%)	33/359 (9.2%)	238/2,726 (8.7%)	0.772
Device therapy	532/3,085 (17.2%)	80/359 (22.3%)	452/2,726 (16.6%)	0.007
**History of valve disease**				
Aortic valve stenosis	375/2,608 (14.4%)	52/277 (18.8%)	323/2,331 (13.9%)	0.028
Aortic valve surgery	793/3,085 (25.7%)	101/359 (28.1%)	692/2,726 (25.4%)	0.263
Mitral valve surgery	376/3,085 (12.2%)	40/359 (11.1%)	336/2,726 (12.3%)	0.519
**Risk factors**				
Previous stroke/TIA	337/2,832 (11.9%)	51/312 (16.3%)	286/2,520 (11.3%)	0.010
Previous pulmonary embolism	64/2,802 (2.3%)	14/307 (4.6%)	50/2,495 (2.0%)	0.005
Arterial hypertension	1,483/3,081 (48.1%)	217/358 (60.6%)	1,266/2,723 (46.5%)	<0.001
Previous hemorrhagic events	128/2,802 (4.6%)	23/305 (7.5%)	105/2,497 (4.2%)	0.008
COPD/asthma	315/3,081 (10.2%)	48/358 (13.4%)	267/2,723 (9.8%)	0.034
Chronic renal failure	544/3,083 (17.6%)	79/359 (22.0%)	465/2,724 (17.1%)	0.021
Dialysis	160/544 (29.4%)	15/79 (19.0%)	145/465 (31.2%)	0.028
HIV	31/3,011 (1.0%)	2/349 (0.6%)	29/2,662 (1.1%)	0.572
Hypo/hyperthyroidism	224/2,792 (8.0%)	33/306 (10.8%)	191/2,486 (7.7%)	0.060
Chronic autoimmune disease	106/3,075 (3.4%)	15/357 (4.2%)	91/2,718 (3.3%)	0.406
Current pregnancy	8/3,062 (0.3%)	1/358 (0.3%)	7/2,704 (0.3%)	>0.999
Smoking	750/2,911 (25.8%)	73/330 (22.1%)	677/2,581 (26.2%)	0.108
Intravenous drug dependency	212/3,038 (7.0%)	3/354 (0.8%)	209/2,684 (7.8%)	<0.001
Alcohol abuse	223/2,974 (7.5%)	23/349 (6.6%)	200/2,625 (7.6%)	0.493
Immunosuppressive treatment	104/2,809 (3.7%)	36/307 (11.7%)	68/2,502 (2.7%)	<0.001
Long corticotherapy	126/2,809 (4.5%)	28/307 (9.1%)	98/2,502 (3.9%)	<0.001
Intravenous catheter	248/3,074 (8.1%)	53/358 (14.8%)	195/2,716 (7.2%)	<0.001
Charlson index mean ± SD	3.48 ± 2.92	6.16 ± 3.35	3.13 ± 2.67	<0.001
Antithrombotic treatment on admission	1,686/2,977 (56.6%)	217/340 (63.8%)	1,469/2,637 (55.7%)	0.005
**Other non-cardiac intervention**				
Colonoscopy	90/2,710 (3.3%)	24/295 (8.1%)	66/2,415 (2.7%)	<0.001
Gastrointestinal intervention	102/3,025 (3.4%)	26/351 (7.4%)	76/2,674 (2.8%)	<0.001
Urogenital intervention	87/3,026 (2.9%)	28/352 (8.0%)	59/2,674 (2.2%)	<0.001
Dental procedure	224/2,849 (7.9%)	16/329 (4.9%)	208/2,520 (8.3%)	0.032

*COPD, Chronic obstructive pulmonary disease; HIV, Human Immunodeficiency Virus; IE, Infective endocarditis; TIA, Transient ischemic attack*.

### Clinical and Biological Features

Clinical features are displayed in Supplementary Table 2. For IE cancer patients, significantly less time passed between first symptoms and first hospitalization (23.7 ± 46.4 vs. 30.1 ± 70.6 days; *P* = 0.009), as well as between first hospitalization and suspected IE (9.1 ± 20.1 vs. 9.2 ± 42.5 days; *P* < 0.001) compared to IE patients without cancer. Platelets were significantly lower in the IE cancer group (194.5 vs. 214 K/mm^3^, *P* < 0.001), but otherwise there was no significant difference in biochemistry between groups (data not shown). Blood cultures were positive in 303/359 (84.4%) IE cancer patients (vs. 78.4%, *P* = 0.009). The most frequently identified microorganisms were *S. aureus* in 77/303 (25.4 vs. 31.8%, *P* = 0.024), Enterococci in 72/303 (23.8 vs. 14.8%, *P* < 0.001), and Streptococcus gallolyticus in 33/303 (10.9 vs. 5.9%, *P* = 0.001) IE cancer patients.

### Imaging

Transthoracic echocardiography (TTE) was performed in 93.9% and transoesophageal echocardiography (TOE) in 82.2% IE cancer patients. There were significantly more mitral valve vegetations (39.9 vs. 34.8%, *P* = 0.020), but less tricuspid valve vegetations (5.3 vs. 10.5%, *P* = 0.008) in IE cancer patients. No significant difference in vegetation length was found between IE cancer and cancer-free groups (data not shown).

18F-FDG positron emission tomography/computed tomography was performed in 74 (20.6%) and positive in 55 IE cancer patients. There was 69.1% extra-cardiac uptake, vs. 54.3% in cancer-free IE patients (*P* = 0.042). On multislice CT, there was significantly more perivalvular abscess formation in IE cancer compared to cancer-free IE patients (78.6 vs. 50.5%, *P* = 0.049).

### In-hospital and One-Year Follow-Up Under Treatment

The main in-hospital complications are shown in Supplementary Table 3.

Acute renal failure was the most frequent in hospital complication in IE cancer patients, followed by embolic events and congestive heart failure (CHF).

After 1 year, there was no significant difference in IE recurrence rate (*P* = 0.243) or other complications between groups.

Cancer IE patients were significantly more treated with amoxicillin (35.8 vs. 26.3%; *P* < 0.001), ceftriaxone (36.3 vs. 31.1%; *P* = 0.047) and daptomycin (15.2 vs. 10.6%; *P* = 0.010), but less frequently treated with vancomycin (34.6 vs. 44.9%, *P* < 0.001) compared to cancer-free IE patients.

Following ESC guidelines, theoretical indication for cardiac surgery was not significantly different between both groups (65.5 vs. 69.8%, *P* = 0.091), but was effectively less performed when indicated in IE cancer patients during hospitalization (65.5 vs. 75.0%, *P* = 0.002). The most frequent surgical indication in both groups was infectious (57.4 vs. 64.9%, *P* = 0.018). Reasons for not performing surgery in IE cancer patients were most frequently the surgical risk (80.2 vs. 54.0%, *P* < 0.001), death before surgery (17.3 vs. 22.9%, *P* = 0.260) and patient refusal (16.0 vs. 19.3%, *P* = 0.486), among others.

Death occurred in hospital in 84 (23.4 vs. 16.1%, *P* < 0.001) and at 1-year follow-up in 43 additional IE cancer patients (18.0 vs. 10.2%; *P* < 0.001). Causes of all-cause in-hospital and 1-year mortality are reported in Tables 2, 3, respectively. Predictors of in hospital and 1-year mortality by univariate Cox regression analysis can be found in Supplementary Tables 4, 5, respectively. Predictors of in hospital and 1-year mortality by multivariable analysis in IE cancer patients are shown in Table 4 and Supplementary Table 6, respectively.

**Table 2:** In-hospital mortality in infective endocarditis patients.

	**Total** **(***n*** = 3,085)**	**IE + cancer** **(***n*** = 359)**	**IE – cancer** **(***n*** = 2,726)**	***P*** **-value**
Death	524/3,085 (17.0%)	84/359 (23.4%)	440/2,726 (16.1%)	<0.001
**Cause of death**				
Cardiovascular	149/523 (28.5%)	15/84 (17.9%)	134/439 (30.5%)	0.067
Non-cardiovascular	155/523 (29.6%)	25/84 (29.8%)	130/439 (29.6%)	
Cardiovascular + Non-cardiovascular	190/523 (36.3%)	39/84 (46.4%)	151/439 (34.4%)	
Unknown	29/523 (5.5%)	5/84 (6.0%)	24/439 (5.5%)	
**If cardiovascular:**				
Heart failure	239/339 (70.5%)	40/54 (74.1%)	199/285 (69.8%)	0.530
Arrhythmia	41/339 (12.1%)	3/54 (5.6%)	38/285 (13.3%)	0.108
Cardiac perforation/tamponade	11/339 (3.2%)	4/54 (7.4%)	7/285 (2.5%)	0.080
Acute MI	7/339 (2.1%)	2/54 (3.7%)	5/285 (1.8%)	0.309
Cerebral embolism	41/339 (12.1%)	4/54 (7.4%)	37/285 (13.0%)	0.249
Pulmonary embolism	13/339 (3.8%)	0/54 (0.0%)	13/285 (4.6%)	0.236
Peripheral embolism	3/339 (0.9%)	0/54 (0.0%)	3/285 (1.1%)	>0.999
**If non-cardiovascular:**				
Neoplasia	12/345 (3.5%)	11/64 (17.2%)	1/281 (0.4%)	<0.001
Sepsis	265/345 (76.8%)	38/64 (59.4%)	227/281 (80.8%)	<0.001

*MI, Myocardial infarction*.

**Table 3:** One-year mortality in infective endocarditis patients.

	**Total** **(***n*** = 3,085)**	**IE + cancer** **(***n*** = 359)**	**IE – cancer** **(***n*** = 2,726)**	***P*** **-value**
Death	233/2,108 (11.1%)	43/239 (18.0%)	190/1,869 (10.2%)	<0.001
**Cause of death**				
Cardiovascular	57/233 (24.5%)	6/43 (14.0%)	51/190 (26.8%)	0.240
Non-cardiovascular	65/233 (27.9%)	16/43 (37.2%)	49/190 (25.8%)	
Cardiovascular + Non-cardiovascular	49/233 (21.0%)	9/43 (20.9%)	40/190 (21.1%)	
Unknown	62/233 (26.6%)	12/43 (27.9%)	50/190 (26.3%)	
**If cardiovascular:**				
Heart failure	74/106 (69.8%)	9/15 (60.0%)	65/91 (71.4%)	
Arrhythmia	9/106 (8.5%)	3/15 (20.0%)	6/91 (6.6%)	
Cardiac perforation/tamponade	1/106 (0.9%)	0/15 (0.0%)	1/91 (1.1%)	
Acute MI	7/106 (6.6%)	1/15 (6.7%)	6/91 (6.6%)	
Cerebral embolism	7/106 (6.6%)	2/15 (13.3%)	5/91 (5.5%)	
Pulmonary embolism	5/106 (4.7%)	1/15 (6.7%)	4/91 (4.4%)	
Peripheral embolism	1/106 (0.9%)	0/15 (0.0%)	1/91 (1.1%)	
Other cardiovascular	27/106 (25.5%)	1/15 (6.7%)	26/91 (28.6%)	
**If non-cardiovascular:**				
Neoplasia	22/114 (19.3%)	15/25 (60.0%)	7/89 (7.9%)	
Sepsis	60/114 (52.6%)	7/25 (28.0%)	53/89 (59.6%)	
Other	41/114 (36.0%)	6/25 (24.0%)	35/89 (39.3%)	

*MI, Myocardial infarction*.

**Table 4:** Multivariate Cox regression analysis for in hospital all-cause mortality (1-month period) in IE cancer patients.

	**Hazard ratio**	**95% CI**	***P*** -value*
Creatinine > 2 mg/dl	2.34	[1.29–4.25]	0.005
Chronic Heart Failure	2.16	[1.18–3.95]	0.013
Surgery: Indication – not performed	2.41	[1.20–4.81]	0.013
Surgery: Indication – performed	0.56	[0.25–1.24]	0.151

*Goodness of Fit test: P = 0.50. Concordance = 0.74 – Global Schoenfeld residual test P = 0.21*.

* *P-value corresponds to the results of the Wald test. For indication – surgery performed, the reference is: no indication*.

**Figure 1:**
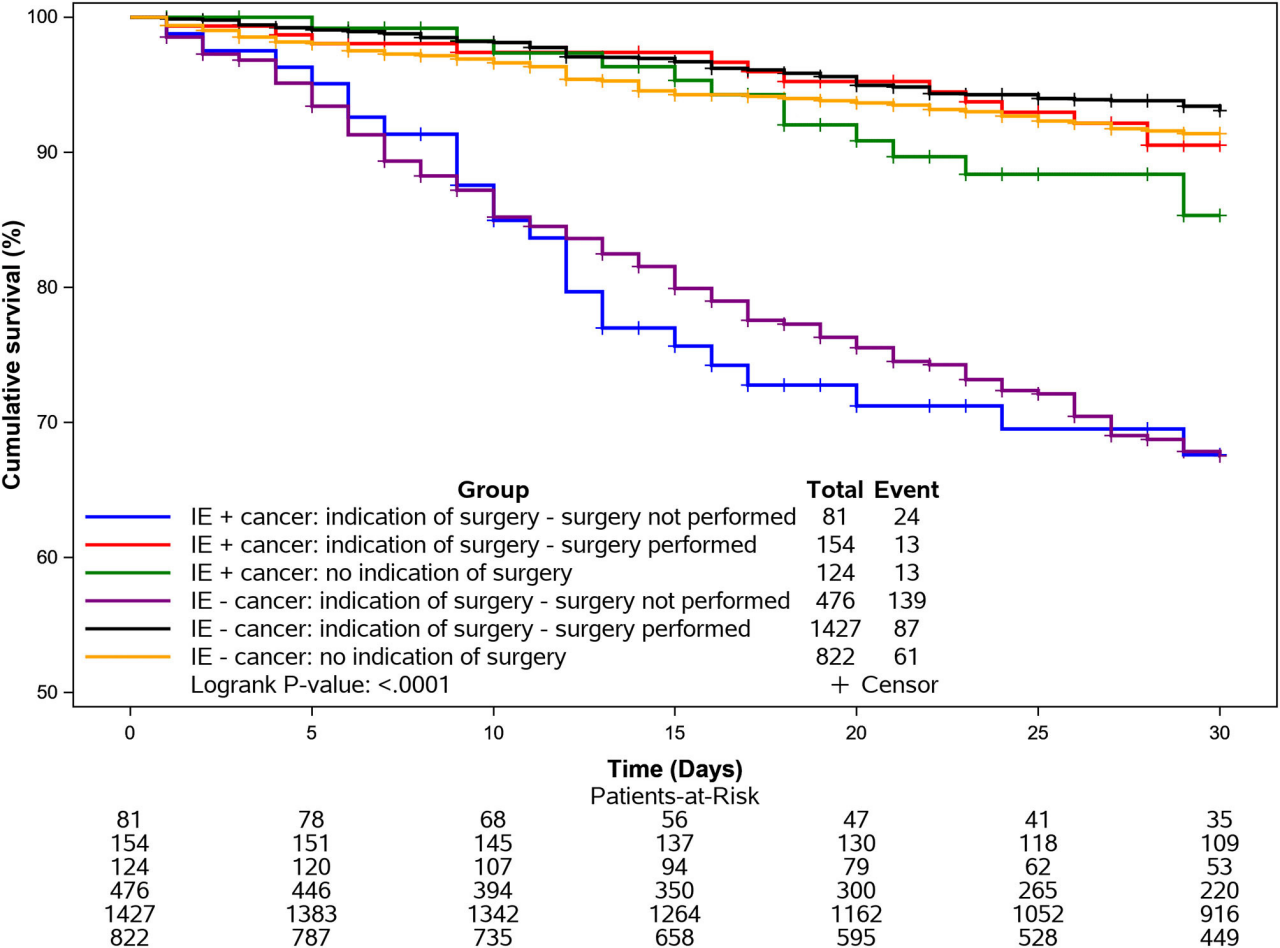
Kaplan-Meier curves for in hospital mortality (1-month) according to cancer and surgery. Mortality was particularly elevated in the IE cancer group when surgery was indicated but not performed.

**Figure 2:**
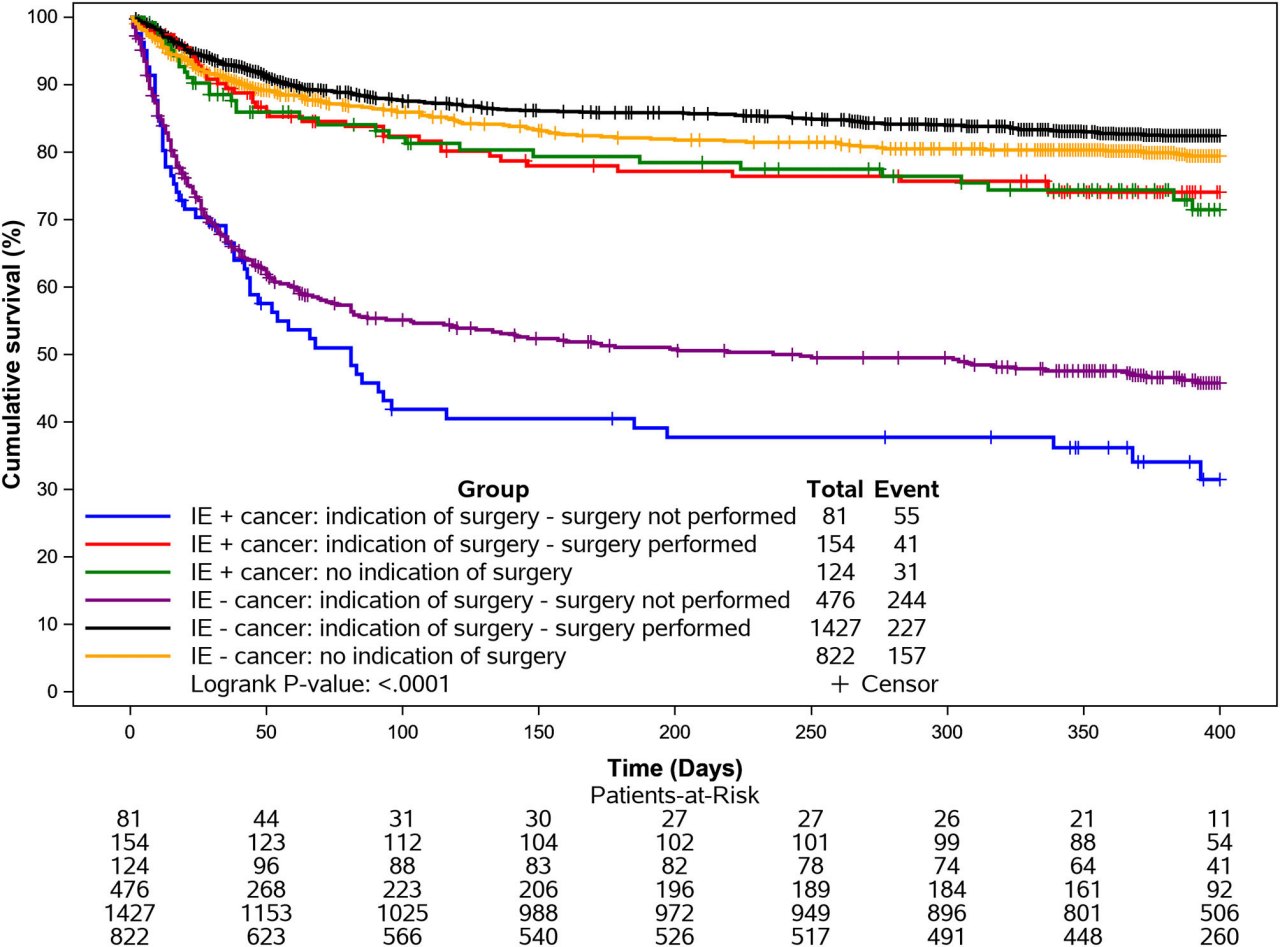
Kaplan-Meier curves for 1-year mortality according to cancer and surgery. Mortality was particularly elevated in the IE cancer group when surgery was indicated but not performed.

Kaplan-Meier survival curves for in hospital and 1-year all-cause mortality according to cancer and adjusted for surgery are shown in Figures 1, 2.

## Discussion

The following key findings arise from the EURO-ENDO analysis regarding cancer in IE patients: 1. Cancer is common in IE patients with a prevalence of 11.6%. 2. IE cancer patients are significantly older, receive more long-term immune-suppressive treatment and have more IV catheters. 3. The most frequently identified microorganisms are *S. aureus* and Enterococci. The source of infection is mainly community-acquired and preceded by non-dental procedures. 4. In hospital and long-term mortality is significantly increased and often related to the neoplasia. 5. Theoretical indication for cardiac surgery is not significantly different, but surgery is significantly less performed when indicated in IE cancer patients compared to IE patients without cancer.

### Demographics, Clinical and Microbiological Characteristics of IE Cancer Patients

Cancer is common in IE patients, with a prevalence of 11.6%. Preceding studies have shown a similar prevalence ranging from 5.6 to 17.6% (6, 8). Prostate- and intestinal neoplasms were found most frequently, which is consistent with previous reports (6, 7). The older age of IE cancer patients has been consistently reported in other series (6, 8, 12). IE cancer patients were more often males, as in the cancer-free group. One study found a slightly significant male predominance in IE cancer patients (6), while another was in agreement with this cohort (8). No gender-based differences were found.

IE cancer patients more often had a history of arterial hypertension, ischemic disease, aortic valve stenosis, atrial fibrillation and previous stroke, probably due to older age. There exists an overlap between cancer and cardiovascular disease, with shared biological mechanisms, risk factors and genetic predisposition (13). Cancer patients had a less typical clinical presentation with significantly less fever and new heart murmur compared to cancer-free IE patients. Nevertheless, cancer patients were hospitalized and diagnosed significantly faster, probably due to close follow-up care. There was no significant difference in embolic events at admission between groups, despite significant more antithrombotics use in IE cancer patients. This was probably compensated by the older age and prothrombogenic status in the cancer group.

IE could be a consequence of cancer management, as immunosuppressive therapy, intravenous access and portal catheters were significantly more present in the IE cancer subpopulation, as previously described (6). Nevertheless, the source of infection was mainly community-acquired in this cohort, and comparable to the cancer-free population (74.8 vs. 74.2%, *P* = 0.06). In contrast, previous studies had reported increased nosocomial IE in cancer patients, but the reason for this discrepancy is unclear (6–8, 14). The most frequent preceding non-cardiac interventions performed in IE cancer patients within the last 6 months were non-dental: urogenital and intestinal (including colonoscopy), as previously reported (6, 8). The significantly higher burden of enterococcal IE might be related to the portal of entry, but also to increased age, as seen in the general population (8, 15). As reported in previous studies, *S. aureus* remained the most frequent causative organism (6, 8). These results, combined with low oral Streptococci (8.9 vs. 12.9%, *P* = 0.05) in blood cultures [compared to the general population in the EuroHeart Survey (15%) (16), the 2008 French registry (20.6%) (10), and the International Collaboration on Endocarditis-Prospective Cohort Study (17%) (3)], reinforce the recommendations of the 2015 ESC guidelines regarding the restriction of the use of antibiotic prophylaxis to high risk populations undergoing at-risk dental procedures (10). Conversely, there might be opportunities for IE prevention in invasive urogenital and gastrointestinal procedures in cancer patients (8).

About 16% of cancer patients had culture-negative endocarditis, which is lower than previously reported, but not significantly different compared to the non-cancer group (8, 17). In these cases, non-bacterial thrombotic endocarditis could not be ruled out.

### Imaging

The transformation in the use of imaging techniques observed in the EURO-ENDO population since the publication of the 2015 ESC guidelines was similarly applicable for IE cancer patients (12). 18F-FDG PET/CT showed more extracardiac liver uptake in IE cancer patients compared to non-cancer patients. Unfortunately, it's difficult to differentiate between metastatic lesions, inflammatory foci and embolic lesions related to IE.

### Management and Outcome of IE in Cancer Patients

#### Surgery

Surgery was performed in ~50% of patients, similar to previous surveys (3, 16). Bioprosthetic valves were used in most IE cancer patients (aortic bioprosthesis: 76.3 vs. mechanical 14.0%; mitral bioprosthesis: 41.5 vs. mechanical 18.5%): more than in non-cancer patients (aortic bioprosthesis: 56.2%, *P* < 0.001; mitral bioprosthesis: 37.2%, *P* = 0.003) and much higher than observed in the Euro heart survey, in which mechanical prosthesis were more prominent (74%). This change might be related to older age, the possible need for further surgical procedures and to an increased risk of bleeding in some neoplasms (18). Mitral valve repair techniques were also more frequently used in cancer compared to non-cancer IE patients (40 vs. 23.4%; *P* = 0.003). This might be explained by a selection bias in the IE cancer group that was accepted for surgery, with a lower operative risk and less valvular destruction (19). Indication for surgery during hospitalization was comparable in cancer vs. non-cancer IE patients. However, when indicated, cardiac surgery was effectively less often performed in cancer compared to cancer-free IE patients. Patients of both groups were mainly denied because of high surgical risk or a significant delay leading to death before surgery.

#### Complications

Acute renal failure was the most frequent complication, followed by embolic events and CHF in IE cancer patients. The older IE cancer group had significantly more underlying chronic renal failure.

There was a significant lower incidence of pulmonary embolism in cancer IE patients. This might be explained by reduced IV drug abuse and less tricuspid valve vegetations in the IE cancer group, as well as a higher proportion of antithrombotic treatment. Nevertheless, this was not reflected by a significant reduction in other embolic events between groups at admission or during hospitalization.

CHF and cardiogenic shock occurred significantly more frequently in IE cancer patients, possibly due to the presence of more cardiovascular disease and frailty in this older population.

#### In-hospital and 1-Year Mortality

In-hospital and long-term all-cause mortality was significantly increased in IE cancer patients compared to the non-cancer population. However, there was no significant difference in cardiovascular death between groups. A main driver of all-cause mortality in cancer patients was the neoplasia, especially at 1-year follow-up. This might be explained by the necessity to interrupt the cancer treatment due to IE, as noted in previous studies (6, 8). Mortality was particularly elevated in the IE cancer group when surgery was indicated but not performed, emphasizing the need for early discussion with surgeons within the IE team, as recommended by the ESC guidelines (19).

#### Study Limitations

This sub-analysis has the same inherent limitations as the EURO-ENDO registry, particularly selection bias as the majority of patients (88.2%) were enrolled in high-level centers in western Europe. Moreover, the study is unlikely to be a true population-based sample, as it was based on voluntary participation and thus it is unsure whether all centers included their patients consecutively and prospectively (12). As a consequence, the true prevalence of cancer in IE patients remains uncertain. As this study was selected from IE patients and not cancer patients, we are also unable to provide incidence data on IE in cancer patients. Moreover, all cancer types might not be appropriately represented, details are missing about cancer characteristics (history, stage, active, or previous treatment) and further investigations are warranted in the occurrence of IE in solid vs. non-solid (e.g., hematological) malignancies, as well as the proportion of metastatic cancer which could influence mortality (8). Moreover, the influence of cancer treatment cessation on mortality should also be taken into consideration. The reason for denial of surgery should be more thoroughly investigated in future studies of IE cancer patients. Clinical reasons could range from a high age, frailty, comorbidities, expected poor prognosis from the underlying malignancy to significant immunosuppression which might render surgery either futile or risky. Moreover, the valvular heart disease guidelines are not specifically written for patients with co-existent malignancies.

As cancer plays a major ponderation in the Charlson score, a sub analysis using an adjusted Charlson score excluding cancer is merited. Additionally, data regarding the occurrence of newly discovered cancer in IE patients, e.g., colon cancer diagnosed by colonoscopy, is absent in this registry. It has been suggested that IE could be an early marker or consequence of occult cancer, particularly that of gastrointestinal or urinary origin (6, 7, 20). Finally, it would be of interest to relate preceding invasive procedures for different types of solid cancers to the bacterial etiologies of IE. These limitations were counterbalanced by the high number of enrolled patients, the quality of CRF completion, and representation of a wide range of both university and non-academic hospitals in many countries around the world.

## Conclusion

This is a large, observational cohort of IE patients with cancer. It provides new insights concerning the contemporary profile, management and clinical outcomes of IE cancer patients. Given the paucity of randomized and large-scale observational data in IE patients with cancer, this registry offers a unique perspective on the current care of IE cancer patients across a wide range of countries.

## Data Availability Statement

The original contributions presented in the study are included in the article/Supplementary Materials, further inquiries can be directed to the corresponding author.

## Ethics Statement

The studies involving human participants were reviewed and approved by Argentina: Comité de Ética de la Investigación, Hospital Italiano de la Plata; Comité de Ética en Investigación, Hospital El Cruce, Florencio Varela; Comite de Investigaciones Médicas, Instituto de Cardiologia de Corrientes JF Cabral; Belgium: Comité d'Ethique hospitalo-facultaire, Université Catholique de Louvain; Comité Local d'Ethique Hospitalier (O.M. 007), Centre Hospitalier Universitaire Saint Pierre, Bruxelles; Commissie Medische Ethiek (O.G. 016), Universitair Ziekenhuis Brussel; Brazil: Comitê de Ética em Pesquisa da Universidade Federal de São Paulo; Comitê de Ética em Pesquisa do Hospital de Messejana, Fortaleza; Comitê de Ética em Pesquisa do Hospital Israelita Albert Einstein, São Paulo; Comitê de Ética em Pesquisa, Faculdade de Medicina de Marília; Comitê de Ética em Pesquisa, Instituto de Cardiologia, Fundacão Universitaria de Cardiologia, Porto Alegre; Comitê de Ética em Pesquisa, Universidad Federal de Minas Gerais; Canada: Comité d'éthique de la recherche de l'Institut Universitaire de Cardiologie et de Pneumologie de Québec, Université Laval; Comité d'éthique de la recherche du Centre Intégré Universitaire de Santé et de Services Sociaux du Nord-de-l'Île-de-Montréal; Czech Republic: Etická komise, Fakultní nemocnice Hradec Králové; Etická komise, Všeobecné fakultní nemocnice v Praze; France: Direction générale de la recherche et de l'innovation, Comité Consultatif sur le traitement de l'information en matière de Recherche dans le domaine de la Santé; Germany: Ethik-Kommission an der Medizinishen Fakultät der Universität Leipzig; Ethikkommission der Universität Leipzig, Ethikkommission der Medizinischen Fakultät des Ruhr-Universität Bochum; Ethikkommission der Universität Leipzig, Ethik-Kommission des FB Medizin; Greece: Laiko General Hospital Managing Committee, Athens; Scientific Council of the University Hospital of Ioannina; India: Medanta Instututional Ethics Committee, Medanta the Medicity, Gurgon; Sengupta Hospital & Research Institute Ethics Committee; Iran: University/Regional Research Ethics Committee, Rajaie Cardiovascular Medical and Research Center; Italy: Comitato Etico Campania Sud, Azienda Sanitaria Locale Napoli 3 Sud; Comitato Etico dell'Ospedale San Raffaele, Istituto di Ricovero e Cura a Carattere Scientifico, Milano; Comitato Etico Milano Area 3, Azienda Socio Sanitaria Territoriale Grande Ospedale Metropolitano Niguarda; Comitato Etico Provinciale, Azienda Ospedaliero-Universitaria di Modena; Comitato Etico Regionale per la Sperimentazione Clinica della Provincia di Padova,; Comitato Etico Regionale per la Sperimentazione Clinica della Toscana, Area Vasta Centro, Azienda Ospedaliero-Universitaria Careggi Firenze; Comitato Etico Regionale per la Sperimentazione Clinica della Toscana, Area Vasta Sud Est, Azienda Ospedaliero-Universitaria Senese; Japan: Nagoya City University Internal Review Board for Clinical Studies; Korea: Institutional Review Board, Asan Medical Center; Institutional Review Board, Samsung Medical Center, Seoul; Lithuania: Lietuvos Bioetikos Komitetas, National Ethics Committee, Vilnius; Moldova: Comitetului de Etica a Cercetarii, N.Testemitanu, State University of Medicine and Pharmacy, Chisinau; Netherlands: Medical Ethics Review Committee of VU University Medical Center, Amsterdam; Portugal: Comissão Nacional de Protecção de Dados, Comissão de Ética para Saude de Centro Hospitalar Universitário de Lisboa Central; Romania: Comisia de Etica a Institutului Inimii de Urgenta pentru Boli Cardiovasculare Niculae Stancioiu, Cluj-Napoca; Comisia de Etica in Cercetare-Dezvoltare, Institutului de Boli Cardiovasculare, University of Medicine & Pharmacy, Timisoara; Russia: Local Ethics Committee City Clinical Hospital (named after V. V. Vinogradov), City Clinical Hospital 64 Moscow; Serbia: Etičkog odbora KBC Zemun, Clinical Hopital Center Zemun, Belgrade; Singapore: National Healthcare Group Domain Specific Review Board, National University Heart Centre Singapore; Spain: Comite de Etica de la Investigation Del Hospital Univesitario Ramon Y Cajal; Comité Ético de Investigación Clínica de Cantabria; Comité Ético de Investigación Clínica de Euskadi; Comité Ético de Investigación Clínica del Hospital Clínico de Barcelona; Comité Ético de Investigación Clínica del Hospital Universitari de Tarragona; Comité Ético de Investigación Clínica del Hospital Universitari Vall d'Hebron de Barcelona; Turkey: Baskent University Institutional Review Board and Ethics Committee, Ankara; United Arab Emirates: Dubai Scientific Research Ethics Committee; United Kingdom: London-Chelsea Research Ethics Committee; United States: IRB #1 Washington, MedStar Heart and Vascular Institute, Washington Hospital Center. The patients/participants provided their written informed consent to participate in this study.

## Author Contributions

All authors listed have made a substantial, direct and intellectual contribution to the work, and approved it for publication.

## Funding

This work was supported by Abbott Vascular Int. (2011–2021), Amgen Cardiovascular (2009–2018), AstraZeneca (2014–2021), Bayer AG (2009–2018), Boehringer Ingelheim (2009–2019), Boston Scientific (2009–2012), the Bristol Myers Squibb and Pfizer Alliance (2011–2019), Daiichi Sankyo Europe GmbH (2011–2020), the Alliance Daiichi Sankyo Europe GmbH and Eli Lilly and Company (2014–2017), Edwards (2016–2019), Gedeon Richter Plc. (2014–2016), Menarini Int. Op. (2009–2012), MSD-Merck & Co. (2011–2014), Novartis Pharma AG (2014–2020), ResMed (2014–2016), Sanofi (2009–2011), SERVIER (2009–2021), and Vifor (2019–2022).

## Conflict of Interest

BI reports personal fees from Edwards Lifesciences, other from Boehringer Ingelheim outside the submitted work. AM reports personal fees from Bayer, personal fees from Fresenius, personal fees from Novartis outside the submitted work. The remaining authors declare that the research was conducted in the absence of any commercial or financial relationships that could be construed as a potential conflict of interest.

## Publisher's Note

All claims expressed in this article are solely those of the authors and do not necessarily represent those of their affiliated organizations, or those of the publisher, the editors and the reviewers. Any product that may be evaluated in this article, or claim that may be made by its manufacturer, is not guaranteed or endorsed by the publisher.

## Abbreviations

CHF: Congestive heart failure
COPD: Chronic obstructive pulmonary disease
CT: Computed tomography
IE: Infective endocarditis
MI: Myocardial infarction
MRI: Magnetic resonance imaging
TIA: Transient ischemic attack
TOE: Transoesophageal echocardiography
TTE: Transthoracic echocardiography.
