# Supplementary material for: Cancer and Infective Endocarditis: Characteristics and Prognostic Impact
Source: Front Cardiovasc Med. 2021 Nov 11;8:766996. doi: 10.3389/fcvm.2021.766996 (PMC8631931; doi:10.3389/fcvm.2021.766996)
Supplement: Supplementary file 1 [file Table_1.DOCX]

Supplementary Material

***Online Table 1.*** Type and age of cancer in infective endocarditis patients.

| **Type of cancer** | **All (n = 359)** |
| --- | --- |
| Prostate | 63 / 307 (20.5%) |
| Liver | 11 / 307 (3.6%) |
| Bowel | 50 / 307 (16.3%) |
| Uterus | 16 / 307 (5.2%) |
| Leukaemia | 14 / 315 (4.4%) |
| Spleen | 1 / 307 (0.3%) |
| Bladder | 23 / 307 (7.5%) |
| Breast | 37 / 307 (12.1%) |
| CNS | 2 / 307 (0.7%) |
| Lymphoma | 24 / 315 (7.6%) |
| Lung | 17 / 307 (5.5%) |
| Kidney | 12 / 307 (3.9%) |
| Brain | 6 / 307 (2.0%) |
| Myeloma | 7 / 307 (2.3%) |
| Other | 87 / 307 (28.3%) |
| **Age of cancer (years)** |  |
| N | 251 |
| Mean ± SD | 6.94 ±7.85 |
| Median (IQR) | 4.0 (1.0-11.0) |
| <= 2 years | 108 / 251 (43.0%) |
| ]2 - 5] years | 30 / 251 (12.0%) |
| ]5 - 10] years | 50 / 251 (19.9%) |
| ]10 - 15] years | 29 / 251 (11.6%) |
| > 15 years | 34 / 251 (13.5%) |

Age of cancer (years) is defined as the time between the diagnosis of cancer (year) and the diagnosis of infective endocarditis (year).

***Online Table 2***. Clinical presentation of infective endocarditis patients.

|  | Total  (n = 3085) | IE + cancer  (n =359) | IE – cancer  (n = 2726) | P-value |
| --- | --- | --- | --- | --- |
| **Signs and symptoms** | |  |  |  |
| Fever | 2357 / 3037 (77.6%) | 255 / 352 (72.4%) | 2102 / 2685 (78.3%) | 0.013 |
| Cough | 514 / 3037 (16.9%) | 39 / 352 (11.1%) | 475 / 2685 (17.7%) | 0.002 |
| Dizziness | 324 / 3037 (10.7%) | 33 / 352 (9.4%) | 291 / 2685 (10.8%) | 0.403 |
| Cerebrovascular accident | 206 / 3037 (6.8%) | 22 / 352 (6.3%) | 184 / 2685 (6.9%) | 0.672 |
| Chest pain | 245 / 3037 (8.1%) | 19 / 352 (5.4%) | 226 / 2685 (8.4%) | 0.051 |
| Shortness of breath | 1004 / 3037 (33.1%) | 91 / 352 (25.9%) | 913 / 2685 (34.0%) | 0.002 |
| Syncope | 79 / 3037 (2.6%) | 13 / 352 (3.7%) | 66 / 2685 (2.5%) | 0.171 |
| Cardiac murmur | 1984 / 3081 (64.4%) | 211 / 358 (58.9%) | 1773 / 2723 (65.1%) | 0.022 |
| Congestive heart failure | 834 / 3085 (27.0%) | 102 / 359 (28.4%) | 732 / 2726 (26.9%) | 0.532 |
| Cardiogenic shock | 62 / 2809 (2.2%) | 10 / 307 (3.3%) | 52 / 2502 (2.1%) | 0.185 |
| Septic shock | 200 / 3084 (6.5%) | 26 / 359 (7.2%) | 174 / 2725 (6.4%) | 0.535 |
| Osler’s nodes | 60 / 3085 (1.9%) | 3 / 359 (0.8%) | 57 / 2726 (2.1%) | 0.105 |
| Janeway lesions | 107 / 3085 (3.5%) | 6 / 359 (1.7%) | 101 / 2726 (3.7%) | 0.048 |
| Roth spots | 44 / 3021 (1.5%) | 5 / 348 (1.4%) | 39 / 2673 (1.5%) | 0.974 |
| **Complications at admission** | |  |  |  |
| Abscess | 358 / 3085 (11.6%) | 36 / 359 (10.0%) | 322 / 2726 (11.8%) | 0.321 |
| Spondylitis | 167 / 3085 (5.4%) | 27 / 359 (7.5%) | 140 / 2726 (5.1%) | 0.061 |
| Embolic events | 787 / 3085 (25.5%) | 85 / 359 (23.7%) | 702 / 2726 (25.8%) | 0.397 |
| Pulmonary | 195 / 787 (24.8%) | 10 / 85 (11.8%) | 185 / 702 (26.4%) | 0.003 |
| Cerebral | 347 / 787 (44.1%) | 34 / 85 (40.0%) | 313 / 702 (44.6%) | 0.421 |
| Splenic | 176 / 787 (22.4%) | 24 / 85 (28.2%) | 152 / 702 (21.7%) | 0.169 |
| Coronary | 22 / 787 (2.8%) | 5 / 85 (5.9%) | 17 / 702 (2.4%) | 0.078 |
| Renal | 75 / 787 (9.5%) | 9 / 85 (10.6%) | 66 / 702 (9.4%) | 0.725 |
| Hepatic | 17 / 787 (2.2%) | 0 / 85 (0.0%) | 17 / 702 (2.4%) | 0.241 |
| Peripheral | 91 / 787 (11.6%) | 11 / 85 (12.9%) | 80 / 702 (11.4%) | 0.674 |
| Haemorrhagic Stroke | 67 / 3085 (2.2%) | 5 / 359 (1.4%) | 62 / 2726 (2.3%) | 0.281 |

IE, Infective endocarditis.

***Online Table 3***. In-hospital complications under therapy in infective endocarditis patients.

|  | Total  (n = 3085) | IE + cancer  (n =359) | IE – cancer  (n = 2726) | P-value |
| --- | --- | --- | --- | --- |
| Complications under therapy |  |  |  |  |
| Embolic events | 635 / 3085 (20.6%) | 78 / 359 (21.7%) | 557 / 2726 (20.4%) | 0.569 |
| Pulmonary | 170 / 3085 (5.5%) | 9 / 359 (2.5%) | 161 / 2726 (5.9%) | 0.008 |
| Cerebral | 279 / 3085 (9.0%) | 39 / 359 (10.9%) | 240 / 2726 (8.8%) | 0.201 |
| TIA | 26 / 279 (9.3%) | 4 / 39 (10.3%) | 22 / 240 (9.2%) | 0.770 |
| Stroke | 166 / 279 (59.5%) | 20 / 39 (51.3%) | 146 / 240 (60.8%) | 0.260 |
| Spleen | 136 / 3085 (4.4%) | 15 / 359 (4.2%) | 121 / 2726 (4.4%) | 0.821 |
| Coronary | 20 / 3085 (0.6%) | 4 / 359 (1.1%) | 16 / 2726 (0.6%) | 0.280 |
| Renal | 58 / 3085 (1.9%) | 10 / 359 (2.8%) | 48 / 2726 (1.8%) | 0.179 |
| Hepatic | 11 / 3085 (0.4%) | 4 / 359 (1.1%) | 7 / 2726 (0.3%) | 0.031 |
| Peripheral | 60 / 3085 (1.9%) | 6 / 359 (1.7%) | 54 / 2726 (2.0%) | 0.690 |
| Haemorrhagic Stroke | 79 / 3085 (2.6%) | 12 / 359 (3.3%) | 67 / 2726 (2.5%) | 0.319 |
| Spondylitis | 144 / 3085 (4.7%) | 24 / 359 (6.7%) | 120 / 2726 (4.4%) | 0.054 |
| CHF | 431 / 3085 (14.0%) | 65 / 359 (18.1%) | 366 / 2726 (13.4%) | 0.016 |
| Cardiogenic shock | 188 / 2809 (6.7%) | 31 / 307 (10.1%) | 157 / 2502 (6.3%) | 0.011 |
| Septic shock | 285 / 3085 (9.2%) | 32 / 359 (8.9%) | 253 / 2726 (9.3%) | 0.821 |
| Glomerulonephritis | 87 / 3066 (2.8%) | 12 / 354 (3.4%) | 75 / 2712 (2.8%) | 0.506 |
| Cerebral Haemorrhage | 71 / 3085 (2.3%) | 8 / 359 (2.2%) | 63 / 2726 (2.3%) | 0.922 |
| Mycotic aneurysm | 58 / 3085 (1.9%) | 9 / 359 (2.5%) | 49 / 2726 (1.8%) | 0.352 |
| Acute renal failure | 543 / 3085 (17.6%) | 93 / 359 (25.9%) | 450 / 2726 (16.5%) | <0.001 |
| Persistent fever (>7 days) | 346 / 2809 (12.3%) | 31 / 307 (10.1%) | 315 / 2502 (12.6%) | 0.210 |
| Positive blood cultures after 48h | 408 / 3057 (13.3%) | 57 / 356 (16.0%) | 351 / 2701 (13.0%) | 0.116 |
| Increasing vegetation size | 198 / 3085 (6.4%) | 22 / 359 (6.1%) | 176 / 2726 (6.5%) | 0.812 |
| New abscess | 190 / 3085 (6.2%) | 20 / 359 (5.6%) | 170 / 2726 (6.2%) | 0.622 |
| AV block | 126 / 2809 (4.5%) | 12 / 307 (3.9%) | 114 / 2502 (4.6%) | 0.605 |
| Thrombopenia (<100000) | 210 / 2809 (7.5%) | 32 / 307 (10.4%) | 178 / 2502 (7.1%) | 0.038 |
|  |  |  |  |  |

CHF, Congestive Heart Failure. [a] For qualitative variables, the Monte Carlo estimates of the exact *P*-values are used.

***Online Table 4***. Univariate Cox regression analysis for in all causes of death in hospital (1-month period) in cancer patients

|  | | Univariate analysis | | |
| --- | --- | --- | --- | --- |
|  | Effect* | Hazard Ratio | 95% CI | *P*-value* |
| ESC countries | ESC | 0.87 | [0.37-2.03] | 0.74 |
| Type of centres | High level IE centres | 0.94 | [0.34-2.61] | 0.90 |
| Type of endocarditis | PM/ICD | 0.77 | [0.23-2.52] | 0.65 |
|  | Prosthesis+Repair | 1.24 | [0.69-2.24] | . |
| Type of endocarditis | Definite IE | 0.61 | [0.31-1.19] | 0.15 |
| Source of infection | Non-nosocomial | 1.00 | [0.31-3.27] | 0.92 |
|  | Nosocomial | 1.17 | [0.55-2.45] | . |
| Age (per ten years) |  | 1.18 | [0.91-1.53] | 0.22 |
| Gender | Female | 0.76 | [040-1.43] | 0.40 |
| Charlson index |  | 1.12 | [1.04-1.20] | 0.002 |
| Creatinine >2mg/dl |  | 2.84 | [1.58-5.11] | 0.001 |
| Staph. Aureus |  | 1.20 | [0.62-2.29] | 0.59 |
| CHF |  | 2.69 | [1.51-4.79] | <0.001 |
| Increased Vegetation size |  | 1.16 | [0.42-3.23] | 0.77 |
| Cerebral complication |  | 1.75 | [0.85-3.60] | 0.13 |
| Abscess |  | 0.79 | [0.31-2.00] | 0.62 |
| Indication - Surgery performed | Indication - not performed | 2.94 | [1.50-5.77] | <0.001 |
|  | Indication - performed | 0.67 | [0.31-1.44] | . |
| Transferred from another hospital |  | 0.69 | [0.37-1.28] | 0.24 |
| Heart Failure |  | 2.32 | [1.28-4.19] | 0.005 |
| Previous pulmonary embolism |  | 3.52 | [1.39-8.93] | 0.008 |

**P*-value corresponds to the results of the Wald test. For type of endocarditis: the reference is native; For indication - surgery performed: no indication; For countries: non-ESC; For type of centres: low level IE; For source of infection: community.

***Online Table 5***. Univariate Cox regression analysis for in all causes of death (1-year period) in cancer patients

|  | | Univariate analysis | | |
| --- | --- | --- | --- | --- |
|  | **Effect*** | **Hazard Ratio** | **95% CI** | **P-value*** |
| ESC countries | ESC | 0.82 | [0.48-1.38] | 0.45 |
| Type of centres | High level IE centres | 0.99 | [0.50-1.95] | 0.98 |
| Type of endocarditis | PM/ICD | 0.63 | [0.29-1.38] | 0.51 |
|  | Prosthesis+Repair | 0.99 | [0.67-1.47] | . |
| Type of endocarditis | Definite IE | 0.83 | [0.51-1.34] | 0.45 |
| Source of infection | Non-nosocomial | 1.16 | [0.50-2.69] | 0.05 |
|  | Nosocomial | 1.80 | [1.13-2.86] | . |
| Age (per ten years) |  | 1.01 | [0.86-1.19] | 0.93 |
| Female gender | Female | 0.58 | [0.37-0.89] | 0.01 |
| Charslon index |  | 1.11 | [1.06-1.16] | <0.001 |
| Creatinine >2mg/dl |  | 2.65 | [1.77-3.99] | <0.001 |
| Staph. Aureus |  | 1.70 | [1.15-2.51] | 0.008 |
| CHF |  | 2.01 | [1.34-3.01] | <0.001 |
| Increased Vegetation size |  | 1.24 | [0.63-2.44] | 0.54 |
| Cerebral complication |  | 1.97 | [1.22-3.19] | 0.006 |
| Abscess |  | 1.31 | [0.78-2.19] | 0.30 |
| Pseudo aneurysm |  | 1.15 | [0.50-2.61] | 0.74 |
| Fistula |  | 2.04 | [0.90-4.63] | 0.09 |
| Paraprosthetic regurgitation |  | 1.37 | [0.72-2.62] | 0.34 |
| Indication - Surgery performed | Indication - not performed | 3.58 | [2.29-5.59] | <0.001 |
|  | Indication - performed | 0.93 | [0.58-1.51] | . |
| Transferred from another hospital |  | 0.79 | [0.54-1.15] | 0.22 |
| Heart Failure |  | 2.19 | [1.48-3.25] | <0.001 |
| Congenital disease |  | 0.23 | [0.03-1.66] | 0.14 |
| Ischaemic heart disease |  | 1.26 | [0.85-1.88] | 0.25 |
| Dilated cardiomyopathy |  | 0.72 | [0.33-1.54] | 0.39 |
| Hypertrophic cardiomyopathy |  | 1.14 | [0.28-4.62] | 0.85 |
| Known valve murmur |  | 1.27 | [0.86-1.88] | 0.22 |
| Previous stroke/TIA |  | 0.63 | [0.35-1.12] | 0.12 |
| Previous Pulmonary embolism |  | 2.66 | [1.29-5.47] | 0.008 |
| Arterial Hypertension |  | 1.38 | [0.95-2.02] | 0.09 |
| Previous Haemorrhagic events |  | 0.96 | [0.47-1.97] | 0.91 |
| COPD/Asthma |  | 1.24 | [0.76-2.03] | 0.38 |
| Chronic renal failure |  | 2.08 | [1.43-3.04] | <0.001 |
| HIV |  | 1.64 | [0.23-11.74] | 0.62 |
| Hypo/Hyperthyroidism |  | 1.29 | [0.74-2.26] | 0.37 |
| Chronic autoimmune disease |  | 2.23 | [1.16-4.26] | 0.02 |
| Intravascular catheter |  | 1.61 | [0.90-2.88] | 0.11 |
| Current Pregnancy |  | NA | NA | 0.98 |
| Smoking |  | 1.34 | [0.87-2.06] | 0.19 |
| Intravenous drug dependency |  | 0.96 | [0.13-6.90] | 0.97 |
| Immunosuppressive treatment |  | 1.67 | [1.02-2.75] | 0.04 |
| Alcohol abuse |  | 0.87 | [0.38-1.98] | 0.74 |
| Diabetes mellitus |  | 1.39 | [0.94-2.06] | 0.10 |
| Atrial fibrillation |  | 1.19 | [0.81-1.74] | 0.37 |
| Location of endocarditis: Aortic |  | 0.94 | [0.66-1.35] | 0.75 |
| Location of endocarditis: Mitral |  | 1.44 | [1.00-2.06] | 0.05 |
| Location of endocarditis: Tricuspid |  | 1.91 | [1.02-3.54] | 0.04 |
| Location of endocarditis: Pulmonary |  | NA | NA | 0.98 |
| Location of endocarditis: ICD/PM |  | 0.87 | [0.47-1.62] | 0.66 |
| Other location of endocarditis |  | 0.56 | [0.14-2.25] | 0.41 |

**P*-value corresponds to the results of the Wald test. For type of endocarditis: the reference is native; For indication - surgery performed: no indication; For countries: non-ESC; For type of centres: low level IE; For source of infection: community. CHF, Congestive Heart Failure.

***Online Table 6***. Multivariate Cox regression analysis for in all-cause mortality (1-year period) in IE cancer patients.

|  | Hazard Ratio | 95% CI | *P*-value* |
| --- | --- | --- | --- |
| Creatinine >2mg/dl | 2.46 | [1.63-3.70] | <0.001 |
| Chronic Heart Failure | 1.59 | [1.05-2.42] | 0.030 |
| Surgery: Indication - not performed | 3.36 | [2.13-5.32] | <0.001 |
| Surgery: Indication - performed | 0.91 | [0.56-1.48] | 0.710 |

Goodness of Fit test: *P* = 0.96 - Concordance= 0.70 – Global Schoenfeld residual test *P* = 0.70. Effects with a *P* value <0.10 in the univariate analysis are taken into account. Cox analysis is performed with a backward procedure with SLSTAY=0.05. For indication - surgery performed, the reference is: no indication. Only 400 days survival data was taken into account: deaths occurring after 400 days were censored at 401 days.

***Appendix 1***

**EORP Oversight Committee :** C.P. Gale, GB (Chair); B. Beleslin, RS; A. Budaj, PL; O. Chioncel, RO; N. Dagres, DE; N. Danchin, FR; J. Emberson, GB; D. Erlinge, SE; M. Glikson, IL; A. Gray, GB; M. Kayikcioglu, TR; A.P. Maggioni, IT; V.K. Nagy, HU; A. Nedoshivin, RU; A-S. Petronio, IT; J. Roos-Hesselink, NL; L. Wallentin, SE; U. Zeymer, DE.

**Executive Committee:** G. Habib, FR (Chair); P. Lancellotti, BE (Chair); B. Cosyns, BE; E. Donal, FR; P. Erba, IT; B. Iung, FR; A.P. Maggioni, IT; B.A. Popescu, RO; B. Prendergast, GB; P. Tornos, ES.

**EORP Team:** M. Andarala, C. Berle, A. Brunel-Lebecq, E. Fiorucci, C. Laroche, V. Missiamenou, C. Taylor.

**National Coordinators:** N.N. Ali Tatar-Chentir, DZ; M. Al-Mallah, SA; M. Astrom Aneq, SE; G. Athanassopoulos, GR; L.P. Badano, IT; S. Benyoussef, TN; E. Calderon Aranda, MX; N.M. Cardim, PT; K-L. Chan, CA; B. Cosyns, BE; I. Cruz, PT; T. Edvardsen, NO; G. Goliasch, AT; G. Habib, FR; A. Hagendorff, DE; K. Hristova, BG; B. Iung, FR; O. Kamp, NL; D-H. Kang, KR; W. Kong, SG; S. Matskeplishvili, RU; M. Meshaal, EG; M. Mirocevic, ME; A.N. Neskovic, RS; M. Pazdernik, CZ; E. Plonska-Gosciniak, PL; B.A. Popescu, RO; B. Prendergast, GB; M. Raissouni, MA; R. Ronderos, AR; L.E. Sade, TR; A. Sadeghpour, IR; A. Sambola, ES; S. Sengupta, IN; J. Separovic-Hanzevacki, HR; M. Takeuchi, JP; E. Tucay, PH; A.C. Tude Rodrigues, BR; A. Varga, HU; J. Vaskelyte, LT; K. Yamagata, MT; K. Yiangou, CY; H. Zaky, AE.

**Investigators**

**Argentina:***Buenos Aires:* I. Granada, M. Mahia, S. Ressi, F. Nacinovich, A. Iribarren, P. Fernandez Oses, G. Avegliano, E. Filipini, *Corrientes:* R. Obregon, M. Bangher, J. Dho, *La Plata:* L. Cartasegna, M.L. Plastino, V. Novas, C. Shigel, *Florencio Varela:* G. Reyes, M. De Santos, N. Gastaldello, M. Granillo Fernandez, M. Potito, G. Streitenberger, P. Velazco, J.H. Casabé, C. Cortes, E. Guevara, F. Salmo, M. Seijo, **Austria:***Vienna:* F. Weidinger, M. Heger, R. Brooks, C. Stöllberger, C-Y. Ho, L. Perschy, L. Puskas, C. Binder, R. Rosenhek, M. Schneider, M-P. Winter, **Belgium:***Liege:* E. Hoffer, M. Melissopoulou, E. Lecoq, D. Legrand, S. Jacquet, M. Massoz, L. Pierard, S. Marchetta, R. Dulgheru, C. D´ Emal, C. Oury, *Jette:* S. Droogmans, D. Kerkhove, D. Plein, L. Soens, C. Weytjens, A. Motoc, B. Roosens, I. Lemoine, *Edegem:* I. Rodrigus, B. Paelinck, B. Amsel, *Brussels:* P. Unger, D. Konopnicki, C. Beauloye, A. Pasquet, J.L. Vanoverschelde , S. Pierard, D. Vancraeynest, F. Sinnaeve, **Brazil:***Sao Paulo:* J.L. Andrade, K. Staszko, *Porto Alegre:* R. Dos Santos Monteiro, M.H. Miglioranza, D.L. Shuha, *Rio de Janeiro:* M. Alcantara, V. Cravo, L. Fazzio, A. Felix, M. Iso, C. Musa, A.P. Siciliano, *Marilia:* F. Villaca Filho, A. Rodrigues, F. Vilela, J. Braga, R. Silva, D. Rodrigues, L. Silva, S. Morhy, C. Fischer, R. Silva, M. Vieira, T. Afonso, *Fortaleza:* J. Abreu, S.N. Falcao, V.A. Moises, A. Gouvea, F.J. Mancuso, A.C. Souza, C.Y. Silva, G. João, C.S. Abboud, R. Bellio de Mattos Barretto, A. Ramos, R. Arnoni, J.E. Assef, D.J. Della Togna, D. Le Bihan, L. Miglioli, A.P. Romero Oliveira, R. Tadeu Magro Kroll, D. Cortez, *Belo Horizonte:* C.L. Gelape, M.d.C. Peirira Nunes, T.C. De Abreu Ferrari, **Canada:***Ottawa:* K. Hay , *Montreal:*V. Le, M. Page, F. Poulin, C. Sauve, K. Serri, C. Mercure, *Quebec:* J. Beaudoin, P. Pibarot, I.A. Sebag, L.G. Rudski, G. Ricafort, **Croatia:***Zagreb:* B. Barsic, V. Krajinovic, M. Vargovic, D. Lovric, V. Reskovic-Luksic, J. Vincelj, S. Jaksic Jurinjak, **Cyprus:***Nicosia:* V. Yiannikourides, M. Ioannides, C. Pofaides, V. Masoura, **Czech Republic:***Ostrava-Poruba:* J. Pudich, *Prague:* A. Linhart, M. Siranec, J. Marek, K. Blechova, M. Kamenik, *Hradec Kralove:* R. Pelouch, *Zlin:* Z. Coufal, M. Mikulica, M. Griva, E. Jancova, M. Mikulcova, *Olomouc:* M. Taborsky, J. Precek, M. Jecmenova, J. Latal, *Liberec:* J. Widimsky, T. Butta, S. Machacek, *Pilsen:* R. Vancata, *Brno:* J. Spinar, M. Holicka, **Ecuador:***Guayaquil:* F. Pow Chon Long, N. Anzules, A. Bajana Carpio, G. Largacha, E. Penaherrera, D. Moreira, **Egypt:***Mansoura:* E. Mahfouz, E. Elsafty, A. Soliman, Y. Zayed, J. Aboulenein, *Alexandria:* M. Abdel-Hay, A. Almaghraby, M. Abdelnaby, M. Ahmed, B. Hammad, Y. Saleh, H. Zahran, O. Elgebaly, *Zagazig:* A. Saad, M. Ali , A. Zeid, R. El Sharkawy, *Cairo:* A. Al Kholy, R. Doss, D. Osama, H. Rizk, A. Elmogy, M. Mishriky, **France:***Kremlin-Bicêtre:* P. Assayag, S. El Hatimi, *Marseille*: S. Hubert, J-P. Casalta, F. Gouriet, F. Arregle, S. Cammilleri, L. Tessonnier, A. Riberi, *Saint-Etienne:* E. Botelho-Nevers, A. Gagneux-Brunon, R. Pierrard, C. Tulane, S. Campisi, J-F. Fuzellier, M. Detoc, T. Mehalla, *Nantes:* D. Boutoille, A.S. Lecompte, M. Lefebvre, S. Pattier, O. Al Habash, N. Asseray-Madani, C. Biron, J. Brochard, J. Caillon, C. Cueff, T. Le Tourneau, R. Lecomte, M.M. Magali Michel, J. Orain, S. Delarue, M. Le Bras, *Limoges:* J-F. Faucher, V. Aboyans, A. Beeharry, H. Durox, M. Lacoste, J. Magne, D. Mohty, A. David, V. Pradel, *Thonon-les-Bains:* V. Sierra, A. Neykova, B. Bettayeb, S. Elkentaoui, B. Tzvetkov, G. Landry, *Reims:* C. Strady, K. Ainine, S. Baumard, C. Brasselet, C. Tassigny, V. Valente-Pires, M. Lefranc, *Pointe-à-Pitre:* B. Hoen, B. Lefevre, E. Curlier, C. Callier, N. Fourcade, *Brest:* Y. Jobic, S. Ansard, R. Le Berre, F. Le Ven, M-C. Pouliquen, G. Prat, P. Le Roux, *Rouen:* F. Bouchart, A. Savoure, C. Alarcon, C. Chapuzet, I. Gueit, *Amiens:* C. Tribouilloy, Y. Bohbot, F. Peugnet , M. Gun, *Paris:* X. Duval, X. Lescure, E. Ilic-Habensus, *Nancy:* N. Sadoul, C. Selton-Suty, F. Alla, F. Goehringer, O. Huttin, E. Chevalier, *Poitiers:* R. Garcia, V. Le Marcis, *Rennes:* P. Tattevin, E. Flecher, M. Revest, *Besançon:* C. Chirouze, K. Bouiller, L. Hustache-Mathieu, T. Klopfenstein, J. Moreau, D. Fournier, A-S. Brunel, *Créteil:* P. Lim, L. Oliver, J. Ternacle, A. Moussafeur, *Dijon:* P. Chavanet, L. Piroth, A. Salmon-Rousseau, M. Buisson, S. Mahy, C. Martins, S. Gohier, *Noumea:* O. Axler, F. Baumann, S. Lebras, **Germany:***Bad Oeynhausen:* C. Piper, D. Guckel, J. Börgermann, D. Horstkotte, E. Winkelmann, B. Brockmeier, *Leipzig:* D. Grey, *Bonn:* G. Nickenig, R. Schueler, C. Öztürk, E. Stöhr, *Bad Nauheim:* C. Hamm, T. Walther, R. Brandt, A-C. Frühauf, C.T. Hartung, C. Hellner, C. Wild, *Aachen:* M. Becker, S. Hamada, W. Kaestner, *Berlin:* K. Stangl, F. Knebel, G. Baldenhofer, A. Brecht, H. Dreger, C. Isner, F. Pfafflin, M. Stegemann, *Ludwigshafen:* R. Zahn, B. Fraiture, C. Kilkowski, A-K. Karcher, S. Klinger, H. Tolksdorf, **Greece:***Athens:* D. Tousoulis, C. Aggeli, S. Sideris, E. Venieri, G. Sarri, D. Tsiapras, I. Armenis, A. Koutsiari, G. Floros, C. Grassos, S. Dragasis, L. Rallidis, C. Varlamos, *Ioannina:* L. Michalis, K. Naka, A. Bechlioulis, A. Kotsia, L. Lakkas, K. Pappas, C. Papadopoulos, S. Kiokas, A. Lioni, S. Misailidou, J. Barbetseas, M. Bonou, C. Kapelios, I. Tomprou, K. Zerva, *Voula:* A. Manolis, E. Hamodraka, D. Athanasiou, G. Haralambidis, H. Samaras, L. Poulimenos, **Hungary:***Budapest:* A. Nagy, A. Bartykowszki, E. Gara, **India:***Nagpur:* K. Mungulmare, *Gurgaon:* R. Kasliwal, M. Bansal, S. Ranjan, A. Bhan, **Iran:***Tehran:* M. Kyavar, M. Maleki, F. Noohi Bezanjani, A. Alizadehasl, S. Boudagh, A. Ghavidel, P. Moradnejad, H.R. Pasha, B. Ghadrdoost, **Israel:***Jerusalem:* D. Gilon, J. Strahilevitz, M. Wanounou, S. Israel, **Italy:***Bari:* C. d'Agostino, P. Colonna, L. De Michele, F. Fumarola, M. Stante, *Florence:* N. Marchionni, V. Scheggi, B. Alterini, S. Del Pace, P. Stefano, C. Sparano, *Padova:* N. Ruozi, R. Tenaglia, D. Muraru , *Grosseto:* U. Limbruno, A. Cresti, P. Baratta, M. Solari, *Milan:* C. Giannattasio, A. Moreo, B. De Chiara, B. Lopez Montero, F. Musca, C.A. Orcese, F. Panzeri, F. Spano, C.F. Russo, O. Alfieri, M. De Bonis, S. Chiappetta, B. Del Forno, M. Ripa, P. Scarpellini, C. Tassan Din, B. Castiglioni , R. Pasciuta, S. Carletti, D. Ferrara, M. Guffanti, G. Iaci, E. Lapenna, T. Nisi, C. Oltolini, E. Busnardo, U. Pajoro, E. Agricola, R. Meneghin, D. Schiavi, *Salerno:* F. Piscione, R. Citro, R.M. Benvenga, L. Greco, L. Soriente, I. Radano, C. Prota, M. Bellino, D. Di Vece, *Genoa:* F. Santini, A. Salsano, G.M. Olivieri, *Modena:* F. Turrini, R. Messora, S. Tondi, A. Olaru, V. Agnoletto, L. Grassi, C. Leonardi, S. Sansoni, *Turin:* S. Del Ponte, G.M. Actis Dato, A. De Martino, **Japan:***Nagoya:* N. Ohte, S. Kikuchi, K. Wakami, *Tsukuba:* K. Aonuma, Y. Seo, T. Ishizu, T. Machino-Ohtsuka, M. Yamamoto, N. Iida, H. Nakajima, *Tenri:* Y. Nakagawa, C. Izumi, M. Amano, M. Miyake, K. Takahashi, *Osaka:* I. Shiojima, Y. Miyasaka, H. Maeba, Y. Suwa, N. Taniguchi, S. Tsujimoto, *Kobe:* T. Kitai, M. Ota, *Sapporo:* S. Yuda, S. Sasaki, *Tokyo:* N. Hagiwara, K. Yamazaki, K. Ashihara, K. Arai, C. Saitou, S. Saitou, G. Suzuki, *Miyazaki:* Y. Shibata, N. Watanabe, S. Nishino, K. Ashikaga, N. Kuriyama, K. Mahara, T. Okubo, H. Fujimaki, H. Shitan, H. Yamamoto, K. Abe, M. Terada, S. Takanashi, *Tokushima:* M. Sata, H. Yamada, K. Kusunose, Y. Saijo, H. Seno, O. Yuichiro, *Suita:* T. Onishi, F. Sera, S. Nakatani, H. Mizuno, K. Sengoku, **Korea, Republic Of:***Seoul:* S.W. Park, K. Eun Kyoung, L. Ga Yeon, J-w. Hwang, C. Jin-Oh, S-J. Park, L. Sang-Chol, C. Sung-A, S.Y. Jang, R. Heo, S. Lee, J-M. Song, E. Jung, **Lithuania:***Siauliai:* J. Plisiene, A. Dambrauskaite, G. Gruodyte, *Kaunas:*R. Jonkaitiene, V. Mizariene, J. Atkocaityte, R. Zvirblyte, **Luxembourg:***Luxembourg:* R. Sow, A. Codreanu, T. Staub, C. Michaux, E.C.L. De la Vega, L. Jacobs-Orazi, **Malta:***Msida:* C. Mallia Azzopardi, R.G. Xuereb, T. Piscopo, J. Farrugia, M. Fenech, E. Pllaha, C. Vella, D. Borg, R. Casha, **Moldova, Republic Of:***Chisinau:* L. Grib, E. Raevschi, A. Grejdieru, D. Kravcenco, E. Prisacari, E. Samohvalov, S. Samohvalov, N. Sceglova, E. Panfile, L. Cardaniuc, V. Corcea, A. Feodorovici, V. Gaina, L. Girbu, P. Jimbei, G. Balan, I. Cardaniuc, I. Benesco, V. Marian, N. Sumarga, **Montenegro:***Podgorica:* B. Bozovic, N. Bulatovic, P. Lakovic, L. Music, **Netherlands:***Rotterdam:* R. Budde, A. Wahadat, T. Gamela, *Amsterdam:* T. Meijers, *Groningen:* J.P. Van Melle, V.M. Deursen, *Maastricht:* H.J. Crijns, S.C. Bekkers, E.C. Cheriex, M. Gilbers, B.L. Kietselaer, C. Knackstedt, R. Lorusso, S. Schalla, S.A. Streukens, *Utrecht:* S. Chamuleau, M-J. Cramer, A. Teske, T. Van der Spoel, A. Wind, J. Lokhorst, O. Liesbek, H. Van Heusden, *The Hague:* W. Tanis, I. Van der Bilt, J. Vriend, H. De Lange-van Bruggen, E. Karijodikoro, R. Riezebos, E. van Dongen, J. Schoep, V. Stolk, **Norway:***Oslo:* J.T. Offstad, J.O. Beitnes, T. Helle-Valle, H. Skulstad, R. Skardal, **Pakistan:***Karachi:* N. Qamar, S. Furnaz, B. Ahmed, M.H. Butt, M.F. Khanzada, T. Saghir, A. Wahid, **Poland:***Warsaw:* T. Hryniewiecki, P. Szymanski, K. Marzec, M. Misztal-Ogonowska, *Wroclaw:* W. Kosmala, M. Przewlocka-Kosmala, A. Rojek, K. Woznicka, J. Zachwyc, *Bialystok:* A. Lisowska, M. Kaminska, *Lodz:* J.D. Kasprzak, E. Kowalczyk, D.F. Strzecka, P. Wejner-Mik, **Portugal:***Carnaxide:* M. Trabulo, P. Freitas, S. Ranchordas, G. Rodrigues, *Guilhufe:* P. Pinto, C. Queiros, J. Azevedo, L. Marques, D. Seabra, *Lisbon:* L. Branco, J. Abreu, M. Cruz, A. Galrinho, R. Moreira, P. Rio, A.T. Timoteo, M. Selas, V. Carmelo, B. Duque Neves, *Almada:* H. Pereira, A. Guerra, A. Marques, I. Pintassilgo, **Romania:***Timisoara:* M.C. Tomescu, N-M. Trofenciuc, M. Andor, A. Bordejevic, H.S. Branea, F. Caruntu, L.A. Velcean, A. Mavrea, M.F. Onel, T. Parvanescu, D. Pop, A.L. Pop-Moldovan, M.I. Puticiu, L. Cirin, I.M. Citu, C.A. Cotoraci, D. Darabantiu, R. Farcas, I. Marincu, A. Ionac, D. Cozma, C. Mornos, F. Goanta, I. Popescu, *Cluj-Napoca:* R. Beyer, R. Mada, R. Rancea, R. Tomoaia, H. Rosianu, C. Stanescu, **Russian Federation:***Moscow:* Z. Kobalava, J. Karaulova, E. Kotova, A. Milto, A. Pisaryuk, N. Povalyaev, M. Sorokina, **Saudi Arabia:***Jeddah:* J. Alrahimi, A. Elshiekh, *Riyadh:* A. Jamiel, A. Ahmed, N. Attia, **Serbia:***Belgrade:* B. Putnikovic, A. Dimic, B. Ivanovic, S. Matic, D. Trifunovic, J. Petrovic, D. Kosevic, I. Stojanovic, I. Petrovic, P. Dabic, P. Milojevic, *Sremska Kamenica:* I. Srdanovic, S. Susak, L. Velicki, A. Vulin, M. Kovacevic, A. Redzek, M. Stefanovic, **Singapore:***Singapore:* T.C. Yeo, W. KF Kong, K.K. Poh, **Spain:***Madrid:* I. Vilacosta, C. Ferrera, C. Olmos, M. Abd El- Nasser , *Vigo - Pontevedra:* F. Calvo Iglesias, E. Blanco-Gonzalez, M. Bravo Amaro, E. Lopez-Rodriguez, J. Lugo Adan, A.N. Germinas, P. Pazos-Lopez, M. Pereira Loureiro, M.T. Perez, S. Raposeiras-Roubin, S. Rasheed Yas, M-M. Suarez-Varela, F. Vasallo Vidal, *Barcelona:* D. Garcia-Dorado, N. Fernandez-Hidalgo, T. Gonzalez-Alujas, J. Lozano, O. Maisterra, N. Pizzi, R. Rios, *Badalona:* A. Bayes-Genis, L. Pedro Botet, N. Vallejo, C. Llibre, L. Mateu, R. Nunez, D. Quesada, E. Berastegui, *Girona:* D. Bosch Portell, J. Aboal Vinas, X. Albert Bertran, R. Brugada Tarradellas, P. Loma-Osorio Ricon, C. Tiron de Llano, *Valencia:* M.A. Arnau, A. Bel, M. Blanes, A. Osa, *Cordoba:* M. Anguita, F. Carrasco, J.C. Castillo, J.L. Zamorano, J.L. Moya Mur, M. Alvaro, C. Fernandez-Golfin, J.M. Monteagudo, E. Navas Elorza, *Santander:* M.C. Farinas Alvarez, J. Aguero Balbin, J. Zarauza, J.F. Gutierrez-Diez, C. Arminanzas , F. Arnaiz de las Revillas, A. Arnaiz Garcia, M. Cobo Belaustegui, M. Fernandez Sampedro, M. Gutierrez Cuadra, L. Garcia Cuello, C. Gonzalez Rico, *Barakaldo:* R. Rodriguez-Alvarez, J. Goikoetxea , M. Montejo , J.M. Miro, M. Almela, J. Ambrosioni, A. Moreno, E. Quintana, E. Sandoval, A. Tellez, J.M. Tolosana, B. Vidal, C. Falces, D. Fuster, C. Garcia-de-la-Maria, M. Hernandez-Meneses, J. Llopis, F. Marco, I. Ruiz-Zamora, *Tarragona:* A. Bardaji Ruiz, E. Sanz Girgas, G. Garcia-Pardo, M. Guillen Marzo, A. Rodriguez Oviedo, A. Villares Jimenez, **Tunisia:***Sfax:* L. Abid, R. Hammami, S. Kammoun, *Tunis:* M.S. Mourali, F. Mghaieth Zghal, M. Ben Hlima, S. Boudiche, S. Ouali, *La Marsa:* L. Zakhama, S. Antit, I. Slama, **Turkey:***Samsun:* O. Gulel, M. Sahin, *Ankara:* L.E. Sade, E. Karacaglar, *Istanbul:* S. Kucukoglu, O. Cetinarslan, U.Y. Sinan, U. Canpolat, B. Mutlu, H. Atas, R. Dervishova, C. Ileri, **United Arab Emirates:***Dubai:* J. Alhashmi, J. Tahir, P. Zarger, F. Baslib, **United Kingdom:***London:* S. Woldman, L. Menezes, C. Primus, R. Uppal, I. Bvekerwa, *Swindon:* B. Chandrasekaran, A. Kopanska, J. Chambers, J. Hancock, J. Klein, R. Rajani, M.P. Ursi, S. Cannata, R. Dworakowski, A. Fife, J. Breeze, M. Browne-Morgan, M. Gunning, S. Streather, **United States:***Washington:* F.M. Asch, M. Zemedkun, **Uzbekistan:***Tashkent:* B. Alyavi, J. Uzokov.
